# Supplementary material for: The Small RNA Universe of Capitella teleta
Source: Front Mol Biosci. 2022 Feb 25;9:802814. doi: 10.3389/fmolb.2022.802814 (PMC8915122; doi:10.3389/fmolb.2022.802814)
Supplement: Supplementary file 1 [file DataSheet1.ZIP › Supplement/known/CAPTEscaffold_296_17124.pdf]

Provisional ID	:	CAPTEscaffold_296_17124
Score total	:	2104296.9
Score for star read(s)	:	3.9
Score for read counts	:	2104290.6
Score for mfe	:	1.6
Score for randfold	:	-2.2
Score for cons. seed	:	3
Total read count	:	4127487
Mature read count	:	4120806
Loop read count	:	63
Star read count	:	6618

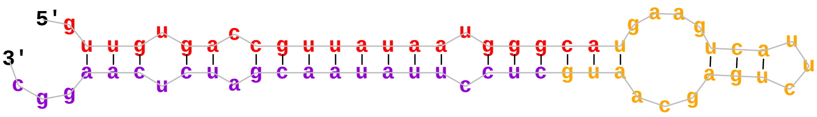

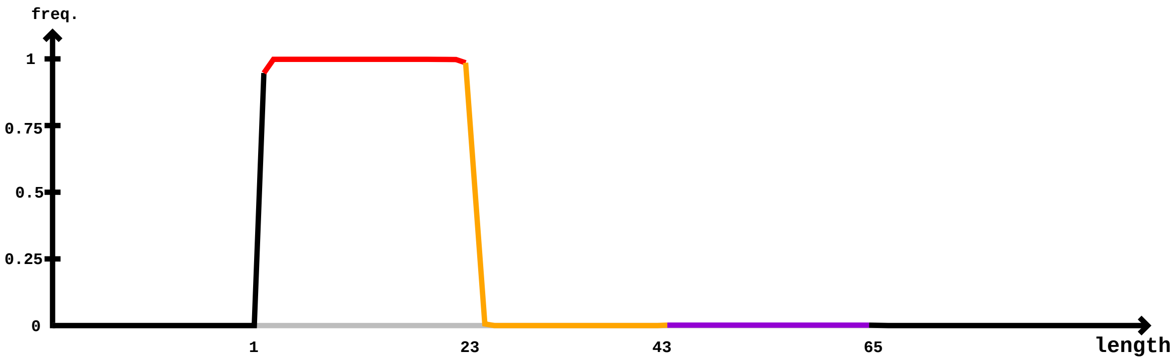


```
                                      Mature                                     Star
5'- gugauaugcccguuccucacguugugaccguuauaaugggcaugaagucauucugagcaaugcuccuuauaacgaucucaaggcgaugggcgcggcacugcgacucuugggc   -3'   obs
    gugauaugcccguuccucacguugugaccguuauaaugggcaugaagucauucugagcaaugcuccuuauaacgaucucaaggcgaugggcgcggcacugcgacucuugggc         exp
    gugauaugcccguuccucacguugugaccguuauaaugggcaugaagucauucugagcaaugcuccuuauaacgaucucaaggcgaugggcgcggcacugcgacucuugggc         known
    (((...((((((((.((.(.(((.((.((((((((.((((((....(((...)))...)))))).)))))))).)).))).).)).))))).)))).)))............   reads   mm   sample
    .................Ucguugugaccguuauaaugggc........................................................................   1       1    seq
    .................Ucguugugaccguuauaaugggca.......................................................................   1       1    seq
    ..................cgAugugaccguuauaaugggca.......................................................................   1       1    seq
    ..................Aguugugaccguuauaaugggca.......................................................................   6       1    seq
    ..................cUuugugaccguuauaaugggca.......................................................................   2       1    seq
    ..................cguugugaccguuauaaugggca.......................................................................   117     0    seq
    ..................cguugugaccguuauaaugAgca.......................................................................   1       1    seq
    ....................guugugaccguuauaGug..........................................................................   1       1    seq
    ....................Uuugugaccguuauaaug..........................................................................   4       1    seq
    ....................guugugaccguuauaaAg..........................................................................   2       1    seq
    ....................guugugaAcguuauaaug..........................................................................   1       1    seq
    ....................guugugaccguuaCaaug..........................................................................   1       1    seq
    ....................guugugGccguuauaaug..........................................................................   2       1    seq
    ....................guugugaccguuauaaug..........................................................................   1117    0    seq
    ....................guugugaccguuauaauA..........................................................................   24      1    seq
    ....................guuguAaccguuauaaug..........................................................................   1       1    seq
    ....................guugugaccgAuauaaug..........................................................................   1       1    seq
    ....................guugugaccguuauaaugA.........................................................................   300     1    seq
    ....................guugugaccguuauaauAg.........................................................................   3       1    seq
    ....................Uuugugaccguuauaaugg.........................................................................   1       1    seq
    ....................guugugaccguuauaaugg.........................................................................   635     0    seq
    ....................guugugaccguuauGaugg.........................................................................   1       1    seq
    ....................guuguAaccguuauaaugg.........................................................................   1       1    seq
    ....................Cuugugaccguuauaaugg.........................................................................   1       1    seq
    ....................guugugaccguuauaaCgg.........................................................................   1       1    seq
    ....................guugugaccguuauaaugU.........................................................................   12      1    seq
    ....................guugugaccguuauaaugC.........................................................................   2       1    seq
    ....................guugugacUguuauaaugg.........................................................................   1       1    seq
    ....................guugugaccguuauaauggg........................................................................   1269    0    seq
    ....................guAgugaccguuauaauggg........................................................................   2       1    seq
    ....................guugugaccguuauaauggA........................................................................   164     1    seq
    ....................gGugugaccguuauaauggg........................................................................   1       1    seq
```

```
                    Mature                                  Star
gugauaugcccguuccucacguugugaccguuauaaugggcaugaagucauucugagcaaugcuccuuauaacgaucucaaggcgaugggcgcggcacugcgacucuugggc
....................guugugaccguuauaauAgg........................................................................ 6      1      seq
....................Nuugugaccguuauaauggg........................................................................ 1      1      seq
....................Uuugugaccguuauaauggg........................................................................ 2      1      seq
....................guugugaccguuauaauggC........................................................................ 92     1      seq
....................guugugaccguuauaaugUg........................................................................ 1      1      seq
....................guugugaccAuuauaauggg........................................................................ 1      1      seq
....................guugugaccguAauaauggg........................................................................ 1      1      seq
....................guugugaccguuauaaugAg........................................................................ 1      1      seq
....................guuguAaccguuauaauggg........................................................................ 1      1      seq
....................guugugacAguuauaauggg........................................................................ 1      1      seq
....................guugugacUguuauaauggg........................................................................ 4      1      seq
....................guugugaccguuauaauggU........................................................................ 2      1      seq
....................gAugugaccguuauaauggg........................................................................ 1      1      seq
....................guugugUccguuauaaugggc....................................................................... 7      1      seq
....................guugugaccguuauaauggUc....................................................................... 6      1      seq
....................guugugaccguuaCaaugggc....................................................................... 3      1      seq
....................guugugaccguuauaaGgggc....................................................................... 1      1      seq
....................guuguAaccguuauaaugggc....................................................................... 36     1      seq
....................Uuugugaccguuauaaugggc....................................................................... 62     1      seq
....................guugGgaccguuauaaugggc....................................................................... 2      1      seq
....................guugugaccguuauaUugggc....................................................................... 7      1      seq
....................Nuugugaccguuauaaugggc....................................................................... 7      1      seq
....................guugugaUcguuauaaugggc....................................................................... 7      1      seq
....................guugugaccgGuauaaugggc....................................................................... 2      1      seq
....................guugugaccguuauGaugggc....................................................................... 5      1      seq
....................guugugaccAuuauaaugggc....................................................................... 10     1      seq
....................guugugaccguuauaaugAgc....................................................................... 37     1      seq
....................guCgugaccguuauaaugggc....................................................................... 11     1      seq
....................guugCgaccguuauaaugggc....................................................................... 4      1      seq
....................guugugaccUuuauaaugggc....................................................................... 4      1      seq
....................guugugaccguuauaaugggG....................................................................... 11     1      seq
....................guAgugaccguuauaaugggc....................................................................... 28     1      seq
....................guugugaccguuGuaaugggc....................................................................... 6      1      seq
....................guugugacAguuauaaugggc....................................................................... 32     1      seq
....................guugugaccguuauaaCgggc....................................................................... 8      1      seq
....................guugugaccguuauaGugggc....................................................................... 7      1      seq
....................guugugaccguCauaaugggc....................................................................... 6      1      seq
....................guugugaccguuauaaugggU....................................................................... 85     1      seq
....................Cuugugaccguuauaaugggc....................................................................... 6      1      seq
....................gAugugaccguuauaaugggc....................................................................... 24     1      seq
....................guugugaccguuauaaugggc....................................................................... 47697  0      seq
....................guugugaccguuauaaugCgc....................................................................... 3      1      seq
....................guugugacUguuauaaugggc....................................................................... 24     1      seq
....................guugugaccguuauUaugggc....................................................................... 1      1      seq
....................guuguCaccguuauaaugggc....................................................................... 2      1      seq
....................guugugaGcguuauaaugggc....................................................................... 2      1      seq
....................guuCugaccguuauaaugggc....................................................................... 6      1      seq
....................guugugaccguuaAaaugggc....................................................................... 4      1      seq
....................guuguUaccguuauaaugggc....................................................................... 1      1      seq
....................guugugaccguuauaauCggc....................................................................... 2      1      seq
....................guugugaccguuauaaugggA....................................................................... 129    1      seq
....................guugugaccCuuauaaugggc....................................................................... 5      1      seq
....................guugugaAcguuauaaugggc....................................................................... 9      1      seq
....................guugugaccgAuauaaugggc....................................................................... 21     1      seq
....................guGgugaccguuauaaugggc....................................................................... 1      1      seq
....................guugugaccguuauaauAggc....................................................................... 98     1      seq
....................guuguNaccguuauaaugggc....................................................................... 2      1      seq
....................guugugaccguuauCaugggc....................................................................... 1      1      seq
....................guugugaccguuauaaugUgc....................................................................... 5      1      seq
....................guugugaccguuauaauUggc....................................................................... 4      1      seq
....................guugugaccguAauaaugggc....................................................................... 13     1      seq
....................guugugGccguuauaaugggc....................................................................... 17     1      seq
....................guuAugaccguuauaaugggc....................................................................... 7      1      seq
....................guugugaccguuauaauggAc....................................................................... 15     1      seq
....................guugugaccguuUuaaugggc....................................................................... 3      1      seq
....................guugAgaccguuauaaugggc....................................................................... 44     1      seq
....................Auugugaccguuauaaugggc....................................................................... 13     1      seq
....................guugugaccguuauaaAgggc....................................................................... 13     1      seq
....................guugugCccguuauaaugggc....................................................................... 4      1      seq
....................gNugugaccguuauaaugggc....................................................................... 1      1      seq
```

```
                    Mature                                    Star
gugauaugcccguuccucacguugugaccguuauaaugggcaugaagucauucugagcaaugcuccuuauaacgaucucaaggcgaugggcgcggcacugcgacucuugggc
....................guugugacGguuauaaugggc.......................................................................    3        1    seq
....................gGugugaccguuauaaugggc.......................................................................    37       1    seq
....................guugugaccguGauaaugggc.......................................................................    2        1    seq
....................guuUugaccguuauaaugggc.......................................................................    2        1    seq
....................guugugaccgCuauaaugggc.......................................................................    5        1    seq
....................gCugugaccguuauaaugggc.......................................................................    3        1    seq
....................guugugaccguuauaauggCc.......................................................................    4        1    seq
....................guugugaccguuaNaaugggca......................................................................    1        1    seq
....................guugugaccguuUuaaugggca......................................................................    431      1    seq
....................Uuugugaccguuauaaugggca......................................................................    5732     1    seq
....................guugugaccguuauGaugggca......................................................................    907      1    seq
....................guugugaccguAauaaugggca......................................................................    803      1    seq
....................guugugaccguuauaaGgggca......................................................................    114      1    seq
....................guugugaccguuauaaugggcC......................................................................    298      1    seq
....................guugGgaccguuauaaugggca......................................................................    178      1    seq
....................guugugaccguuauaaugggca......................................................................    3728069  0    seq
....................guugugacUguuauaaugggca......................................................................    2511     1    seq
....................guugugaccguuauaauCggca......................................................................    279      1    seq
....................guugugaccguuauaauggCca......................................................................    16021    1    seq
....................guugugaccguuauaaugggNa......................................................................    1        1    seq
....................guuguAaccguuauaaugggca......................................................................    2107     1    seq
....................gNugugaccguuauaaugggca......................................................................    174      1    seq
....................guuUugaccguuauaaugggca......................................................................    168      1    seq
....................guugugaccguuCuaaugggca......................................................................    7        1    seq
....................guugugaccguuauaCugggca......................................................................    26       1    seq
....................guugugaccguuauaGugggca......................................................................    490      1    seq
....................guugugaGcguuauaaugggca......................................................................    205      1    seq
....................guuCugaccguuauaaugggca......................................................................    198      1    seq
....................guugugaccguuauaauggAca......................................................................    706      1    seq
....................guugugaccguuauaauggUca......................................................................    371      1    seq
....................guugugaccguuaAaaugggca......................................................................    546      1    seq
....................guugugaccgCuauaaugggca......................................................................    435      1    seq
....................guugugaccguuauaaugggUa......................................................................    663      1    seq
....................guugugaccguuauaaugggAa......................................................................    1072     1    seq
....................guugugCccguuauaaugggca......................................................................    304      1    seq
....................guugugaccAuuauaaugggca......................................................................    671      1    seq
....................guuguUaccguuauaaugggca......................................................................    235      1    seq
....................gAugugaccguuauaaugggca......................................................................    1730     1    seq
....................guugugNccguuauaaugggca......................................................................    1        1    seq
....................guugugGccguuauaaugggca......................................................................    1026     1    seq
....................guugugaccguuauaaugggcG......................................................................    1374     1    seq
....................guuguCaccguuauaaugggca......................................................................    338      1    seq
....................guuAugaccguuauaaugggca......................................................................    625      1    seq
....................guugugaccguCauaaugggca......................................................................    541      1    seq
....................guGgugaccguuauaaugggca......................................................................    68       1    seq
....................guugugaUcguuauaaugggca......................................................................    502      1    seq
....................guugugacGguuauaaugggca......................................................................    243      1    seq
....................guugNgaccguuauaaugggca......................................................................    1        1    seq
....................guAgugaccguuauaaugggca......................................................................    2086     1    seq
....................guugugaccguuauaaugAgca......................................................................    2650     1    seq
....................guugugaccUuuauaaugggca......................................................................    488      1    seq
....................guuguNaccguuauaaugggca......................................................................    7        1    seq
....................guugugaccguuauaaAgggca......................................................................    1323     1    seq
....................guugugaccguuauaauAggca......................................................................    12154    1    seq
....................guugugaccguuauaaugCgca......................................................................    261      1    seq
....................guugugaccCuuauaaugggca......................................................................    231      1    seq
....................Nuugugaccguuauaaugggca......................................................................    1134     1    seq
....................guNgugaccguuauaaugggca......................................................................    24       1    seq
....................guugugaccguuauaauUggca......................................................................    374      1    seq
....................guugugaccguuauaaugggcU......................................................................    2416     1    seq
....................guugugaccNuuauaaugggca......................................................................    2        1    seq
....................gGugugaccguuauaaugggca......................................................................    2090     1    seq
....................guugCgaccguuauaaugggca......................................................................    484      1    seq
....................guugugaccguuaGaaugggca......................................................................    118      1    seq
....................guugugUccguuauaaugggca......................................................................    412      1    seq
....................guugugaccguuauaaCgggca......................................................................    373      1    seq
....................guugugaccguuauaaugggGa......................................................................    538      1    seq
....................guugugaAcguuauaaugggca......................................................................    712      1    seq
....................guugugaccguuaCaaugggca......................................................................    443      1    seq
....................guugugaccguuauaaugUgca......................................................................    364      1    seq
```

Mature **Star**

gugauaugcccguuccucacguugugaccguuauaaugggcaugaagucauucugagcaaugcuccuuauaacgaucucaaggcgaugggcgcggcacugcgacucuugggc			
....................guugugaccguuauaNugggca..	1	1	seq
....................Cuugugaccguuauaaugggca..	830	1	seq
....................guugugaccguuauCaugggca..	20	1	seq
....................guugugaccgGuauaaugggca..	232	1	seq
....................guugugacAguuauaaugggca..	1480	1	seq
....................guugugaccguuauUaugggca..	101	1	seq
....................guugugaccguuauaUugggca..	416	1	seq
....................guugAgaccguuauaaugggca..	3010	1	seq
....................guugugaccguuGuaaugggca..	660	1	seq
....................Auugugaccguuauaaugggca..	813	1	seq
....................guugugaccguGauaaugggca..	111	1	seq
....................guugugaccgAuauaaugggca..	1301	1	seq
....................guCgugaccguuauaaugggca..	640	1	seq
....................gCugugaccguuauaaugggca..	392	1	seq
....................guugugaccguuauaaNgggca..	3	1	seq
....................guugugaccguuauaaugggcaN...	3	1	seq
....................guuAugaccguuauaaugggcau...	1	1	seq
....................guugugaccguAauaaugggcau...	1	1	seq
....................guuCugaccguuauaaugggcau...	1	1	seq
....................guugugaccgAuauaaugggcau...	2	1	seq
....................guugugaccgCuauaaugggcau...	1	1	seq
....................guugugaccguuauaaugggcaG...	11	1	seq
....................guAgugaccguuauaaugggcau...	3	1	seq
....................gAugugaccguuauaaugggcau...	2	1	seq
....................guugugaccCuuauaaugggcau...	1	1	seq
....................guugugaccguuauaaugggcUu...	23	1	seq
....................guugugaccguuauaaugggUau...	4	1	seq
....................guugugaccguuauaaugAgcau...	2	1	seq
....................guugugacUguuauaaugggcau...	2	1	seq
....................guugugaccguuauaaAgggcau...	2	1	seq
....................gNugugaccguuauaaugggcau...	1	1	seq
....................guugugaccguuauaaugggcaC...	28	1	seq
....................guugugaccguuauGaugggcau...	3	1	seq
....................Nuugugaccguuauaaugggcau...	1	1	seq
....................guugugGccguuauaaugggcau...	1	1	seq
....................gGugugaccguuauaaugggcau...	3	1	seq
....................guugugaccguuaAaaugggcau...	1	1	seq
....................guugugaccguuaCaaugggcau...	1	1	seq
....................guugCgaccguuauaaugggcau...	3	1	seq
....................guuguAaccguuauaaugggcau...	2	1	seq
....................guugugaccguuauaaugggAau...	3	1	seq
....................guugugaccguuauaauggAcau...	1	1	seq
....................Auugugaccguuauaaugggcau...	1	1	seq
....................guugAgaccguuauaaugggcau...	5	1	seq
....................guugugCccguuauaaugggcau...	1	1	seq
....................guugugaccguuauaaugggcCu...	3	1	seq
....................guugugaccAuuauaaugggcau...	1	1	seq
....................guugugaccguuauaaGgggcau...	1	1	seq
....................guugugaccguuauaaugggcau...	5274	0	seq
....................guugugaccgGuauaaugggcau...	2	1	seq
....................guugugaccguuauaauAggcau...	18	1	seq
....................guugugUccguuauaaugggcau...	1	1	seq
....................guugugaccguuGuaaugggcau...	2	1	seq
....................guugugaccguuauaaugggcaA...	20376	1	seq
....................Uuugugaccguuauaaugggcau...	12	1	seq
....................guugugacAguuauaaugggcau...	4	1	seq
....................guugugaccguuUuaaugggcau...	2	1	seq
....................guugugaccguuauaaugggcauC..	59	1	seq
....................guuUugaccguuauaaugggcaug..	1	1	seq
....................guugugaccguuauaaugggcaAg..	4	1	seq
....................guugugacUguuauaaugggcaug..	1	1	seq
....................guugugaccguuauaaugAgcaug..	1	1	seq
....................guugAgaccguuauaaugggcaug..	1	1	seq
....................Uuugugaccguuauaaugggcaug..	1	1	seq
....................guugugaccguuauaaugggcauA..	3894	1	seq
....................guugugaccguuauaaugggcauU..	17749	1	seq
....................guugugaccguuauaaugggcaug..	141	0	seq
....................guugugaccguuauaaugggcauUa...	4	1	seq
....................guugugaccguuauaaugggcauga...	2	0	seq
....................guugugaccguuauaaugggcauAa...	7	1	seq

```
                    Mature                                      Star
gugauaugcccguuccucacguugugaccguuauaaugggcaugaagucauucugagcaaugcuccuuauaacgaucucaaggcgaugggcgcggcacugcgacucuugggc
....................guugugaccguuauaaugggcauAaa..................................................................  3     1  seq
.....................uugugaccguuauaaugg.........................................................................  4     0  seq
.....................uugugaccguuauaauggg........................................................................  24    0  seq
.....................uugugaccguuauaauggA........................................................................  7     1  seq
.....................uugugaccguuauaauggCc.......................................................................  1     1  seq
.....................Augugaccguuauaaugggc.......................................................................  5     1  seq
.....................uugCgaccguuauaaugggc.......................................................................  1     1  seq
.....................uugugaccguuauaaugggU.......................................................................  2     1  seq
.....................uugugCccguuauaaugggc.......................................................................  1     1  seq
.....................uugugaccguuauaauggAc.......................................................................  1     1  seq
.....................uugugaccguuauaaugggA.......................................................................  1     1  seq
.....................uugugaccguuauaaugggc.......................................................................  1196  0  seq
.....................uuguAaccguuauaaugggc.......................................................................  1     1  seq
.....................Nugugaccguuauaaugggc.......................................................................  1     1  seq
.....................uugugaccguuauaauAggc.......................................................................  4     1  seq
.....................uugugaccgAuauaaugggc.......................................................................  1     1  seq
.....................uAgugaccguuauaaugggc.......................................................................  1     1  seq
.....................uugugacUguuauaaugggc.......................................................................  1     1  seq
.....................Gugugaccguuauaaugggc.......................................................................  2     1  seq
.....................uugugaccguuauaaugAgc.......................................................................  3     1  seq
.....................uugugaccUuuauaaugggc.......................................................................  1     1  seq
.....................uugugaccguuauaaNgggca......................................................................  1     1  seq
.....................uugugaccguuauGaugggca......................................................................  46    1  seq
.....................uugugaccguuauaaAgggca......................................................................  89    1  seq
.....................uugugaccAuuauaaugggca......................................................................  45    1  seq
.....................uugugaccgGuauaaugggca......................................................................  7     1  seq
.....................uugugaccguuaCaaugggca......................................................................  21    1  seq
.....................uugugaccguuauaaugggcU......................................................................  176   1  seq
.....................uugugaccguuauaaugggAa......................................................................  86    1  seq
.....................uugugaAcguuauaaugggca......................................................................  88    1  seq
.....................uugugaccguuauaaugCgca......................................................................  10    1  seq
.....................uugugaccguuauaGugggca......................................................................  31    1  seq
.....................uugAgaccguuauaaugggca......................................................................  97    1  seq
.....................uugugaUcguuauaaugggca......................................................................  31    1  seq
.....................uugugaccguuauaaugggGa......................................................................  19    1  seq
.....................uugugaccguuauaaCgggca......................................................................  16    1  seq
.....................uugugaccguuauaaugggcG......................................................................  64    1  seq
.....................uugugaccgCuauaaugggca......................................................................  29    1  seq
.....................uuCugaccguuauaaugggca......................................................................  13    1  seq
.....................uugugaccguuaAaaugggca......................................................................  39    1  seq
.....................uugugaccguuauaauAggca......................................................................  737   1  seq
.....................uuAugaccguuauaaugggca......................................................................  26    1  seq
.....................uugugaccguuauaauggCca......................................................................  1131  1  seq
.....................uugugaccguuauaaugAgca......................................................................  179   1  seq
.....................uugugaGcguuauaaugggca......................................................................  24    1  seq
.....................uuUugaccguuauaaugggca......................................................................  11    1  seq
.....................uugugaccguuauaauggUca......................................................................  23    1  seq
.....................uugugaccguuauaauUggca......................................................................  33    1  seq
.....................uugugaccguuauaCugggca......................................................................  1     1  seq
.....................uugugaccguGauaaugggca......................................................................  7     1  seq
.....................uugugaccguAauaaugggca......................................................................  62    1  seq
.....................uugugaccguuUuaaugggca......................................................................  15    1  seq
.....................uugugaccguCauaaugggca......................................................................  32    1  seq
.....................uugugacGguuauaaugggca......................................................................  5     1  seq
.....................uugugaccCuuauaaugggca......................................................................  8     1  seq
.....................uGgugaccguuauaaugggca......................................................................  5     1  seq
.....................uuguAaccguuauaaugggca......................................................................  132   1  seq
.....................uugugaccguuauCaugggca......................................................................  1     1  seq
.....................uugugaccguuauUaugggca......................................................................  6     1  seq
.....................uugugaccguuGuaaugggca......................................................................  36    1  seq
.....................uugugacAguuauaaugggca......................................................................  76    1  seq
.....................Augugaccguuauaaugggca......................................................................  518   1  seq
.....................uugugaccguuauaUugggca......................................................................  23    1  seq
.....................uugugaccguuauaaugUgca......................................................................  27    1  seq
.....................uugugGccguuauaaugggca......................................................................  100   1  seq
.....................uugugaccguuauaaGgggca......................................................................  14    1  seq
.....................uugugaccguuauaauggAca......................................................................  42    1  seq
.....................uugugacUguuauaaugggca......................................................................  150   1  seq
.....................uugCgaccguuauaaugggca......................................................................  30    1  seq
.....................Nugugaccguuauaaugggca......................................................................  49    1  seq
```

```
                    Mature                                    Star
gugauaugcccguuccucacguugugaccguuauaaugggcaugaagucauucugagcaaugcuccuuauaacgaucucaaggcgaugggcgcggcacugcgacucuugggc
.....................uugugaccgAuauaaugggca......................................................................  92      1  seq
.....................uugugaccguuauaaugggUa......................................................................  30      1  seq
.....................uugugaccguuauaaugggca......................................................................  198118  0  seq
.....................uugugCccguuauaaugggca......................................................................  21      1  seq
.....................uuguUaccguuauaaugggca......................................................................  11      1  seq
.....................uugugUccguuauaaugggca......................................................................  26      1  seq
.....................uugugaccguuauaaugggcC......................................................................  29      1  seq
.....................uCgugaccguuauaaugggca......................................................................  30      1  seq
.....................uugugaccguuaGaaugggca......................................................................  17      1  seq
.....................uAgugaccguuauaaugggca......................................................................  96      1  seq
.....................uugugaccguuauaauCggca......................................................................  24      1  seq
.....................uuguCaccguuauaaugggca......................................................................  17      1  seq
.....................Cugugaccguuauaaugggca......................................................................  25      1  seq
.....................Gugugaccguuauaaugggca......................................................................  187     1  seq
.....................uNgugaccguuauaaugggca......................................................................  8       1  seq
.....................uugugaccUuuauaaugggca......................................................................  12      1  seq
.....................uugGgaccguuauaaugggca......................................................................  18      1  seq
.....................uugugaUcguuauaaugggcau.....................................................................  1       1  seq
.....................uugugaccguuauaaugggcaA.....................................................................  146     1  seq
.....................uugugaccguuauaaugggUau.....................................................................  1       1  seq
.....................uugugaccguuauaaugUgcau.....................................................................  1       1  seq
.....................uuguUaccguuauaaugggcau.....................................................................  1       1  seq
.....................uugugacUguuauaaugggcau.....................................................................  4       1  seq
.....................uugugaccguuauaauggCcau.....................................................................  1       1  seq
.....................uugugaccguuauaaugggGau.....................................................................  1       1  seq
.....................uugugaccguuaCaaugggcau.....................................................................  1       1  seq
.....................Cugugaccguuauaaugggcau.....................................................................  1       1  seq
.....................Gugugaccguuauaaugggcau.....................................................................  2       1  seq
.....................uugugaccguAauaaugggcau.....................................................................  1       1  seq
.....................uugugaccguuauaaCgggcau.....................................................................  1       1  seq
.....................uugugaAcguuauaaugggcau.....................................................................  2       1  seq
.....................uAgugaccguuauaaugggcau.....................................................................  4       1  seq
.....................uugugaccguuauaauAggcau.....................................................................  13      1  seq
.....................uugugaccguuauaaugggcau.....................................................................  3713    0  seq
.....................uuAugaccguuauaaugggcau.....................................................................  1       1  seq
.....................uugugaccguuaAaaugggcau.....................................................................  1       1  seq
.....................uugugaccgAuauaaugggcau.....................................................................  1       1  seq
.....................uugugaccguuauaaugggcaC.....................................................................  9       1  seq
.....................uugugaccguuauaauUggcau.....................................................................  1       1  seq
.....................uugugaccguuauaaAgggcau.....................................................................  1       1  seq
.....................uugAgaccguuauaaugggcau.....................................................................  1       1  seq
.....................uugugaccguuauaaugAgcau.....................................................................  4       1  seq
.....................uuguAaccguuauaaugggcau.....................................................................  5       1  seq
.....................uugugaccguuauaaugggAau.....................................................................  2       1  seq
.....................uugugaccguuauaauggAcau.....................................................................  2       1  seq
.....................Augugaccguuauaaugggcau.....................................................................  12      1  seq
.....................uugugGccguuauaaugggcau.....................................................................  1       1  seq
.....................uugugaccguuauaaugggcUu.....................................................................  3       1  seq
.....................uugugaccguCauaaugggcau.....................................................................  1       1  seq
.....................uugugaccguuUuaaugggcau.....................................................................  1       1  seq
.....................uugugaccguuauaaGgggcau.....................................................................  1       1  seq
.....................uugugaccguuauaaugggcaG.....................................................................  1       1  seq
.....................uugugaccguuauGaugggcau.....................................................................  2       1  seq
.....................uugugaccguuauaaugggcauU....................................................................  2582    1  seq
.....................uugugaccguuauaaugggcauA....................................................................  721     1  seq
.....................uugugaccguuauaaugggcaug....................................................................  15      0  seq
.....................uugugaccguuauaaugggcauN....................................................................  1       1  seq
.....................uugugaccguuauaaugggcauC....................................................................  4       1  seq
......................ugugaccguuauaauAggca......................................................................  1       1  seq
......................ugugaccguuauaaugggca......................................................................  103     0  seq
......................ugugaccguuGuaaugggca......................................................................  1       1  seq
......................ugugaUcguuauaaugggca......................................................................  1       1  seq
......................ugugaccguuauaaugggcaug....................................................................  4       0  seq
......................ugugaccguuauGaugggcaug....................................................................  1       1  seq
.......................Uugaccguuauaaugggca......................................................................  21      1  seq
.......................gugaccguuauaaugggca......................................................................  7       0  seq
.......................guUaccguuauaaugggca......................................................................  1       1  seq
.......................gugaccguuauaaugggcaug....................................................................  3       0  seq
........................ugaccguuauaaugggca......................................................................  4       0  seq
..........................accguuauaaugggcaugaaguc...............................................................  1       0  seq
```

Mature Star

```
gugauaugcccguuccucacguugugaccguuauaaugggcaugaagucauucugagcaaugcuccuuauaacgaucucaaggcgaugggcgcggcacugcgacucuugggc
..........................................ugaagucauucugagcaa....................................................  2     0  seq
..........................................ugaagAcauucugagcaaug..................................................  1     1  seq
..........................................ugaagucauucugagcaaug..................................................  54    0  seq
..........................................ugaagucauucugagcaaugA.................................................  1     1  seq
...........................................gaagucauucugagcaau...................................................  5     0  seq
............................................................ugcuccuuauaacgaucucaag..............................  1     0  seq
.............................................................gcuccuuauaacgaucucaag..............................  6     0  seq
.............................................................gcuccuuauaacgaucucaagg.............................  35    0  seq
.............................................................gcuccuuauaacgaucucaaggA............................  1     1  seq
..............................................................cuccuuauaacgaucucaag..............................  66    0  seq
..............................................................cuccuuauaacgGucucaag..............................  1     1  seq
..............................................................Nuccuuauaacgaucucaagg.............................  1     1  seq
..............................................................cuccuuauaUcgaucucaagg.............................  1     1  seq
..............................................................cuccuuaCaacgaucucaagg.............................  1     1  seq
..............................................................cuccuuauaacAaucucaagg.............................  2     1  seq
..............................................................cuccuuauaacUaucucaagg.............................  1     1  seq
..............................................................cuccuuauaacgaucucaagg.............................  538   0  seq
..............................................................cuccuuauaacgaucucaagA.............................  2     1  seq
..............................................................cuccuuauaacgaucAcaagg.............................  1     1  seq
..............................................................cCccuuauaacgaucucaagg.............................  1     1  seq
..............................................................cuccuuauaacgaAcucaaggc............................  1     1  seq
..............................................................cuccuuauaacgaucuUaaggc............................  3     1  seq
..............................................................cuccuAauaacgaucucaaggc............................  3     1  seq
..............................................................cuccuuauaacgaucucaUggc............................  1     1  seq
..............................................................cuccuuauaacgGucucaaggc............................  2     1  seq
..............................................................cuccuuauaacgaucucaGggc............................  2     1  seq
..............................................................cuccuuauaacgaucucGaggc............................  1     1  seq
..............................................................cuccuuauaacCaucucaaggc............................  1     1  seq
..............................................................cuccuGauaacgaucucaaggc............................  1     1  seq
..............................................................cuUcuuauaacgaucucaaggc............................  3     1  seq
..............................................................cuccuuauaacgaucucaagUc............................  2     1  seq
..............................................................cAccuuauaacgaucucaaggc............................  6     1  seq
..............................................................cuccuuauaGcgaucucaaggc............................  1     1  seq
..............................................................cuccuuauaacgaucucaaggA............................  29    1  seq
..............................................................cuccuuauaacgaucucaaggc............................  3947  0  seq
..............................................................cuccuuauaacAaucucaaggc............................  16    1  seq
..............................................................cuccuuauaacgaucucaaggU............................  29    1  seq
..............................................................cuccuuauaacgauAucaaggc............................  1     1  seq
..............................................................cuccuuauaacgaucucaagCc............................  1     1  seq
..............................................................cuccAuauaacgaucucaaggc............................  6     1  seq
..............................................................Uuccuuauaacgaucucaaggc............................  3     1  seq
..............................................................cucUuuauaacgaucucaaggc............................  3     1  seq
..............................................................cuccuuauaacgaucucaaAgc............................  1     1  seq
..............................................................cuccuuauaacgaucucaaggcU...........................  1692  1  seq
..............................................................cuccuuauaacAaucucaaggcg...........................  1     1  seq
..............................................................cuccuuauaacgaucucaaggcg...........................  8     0  seq
..............................................................cuccuuauaacgaucucaaggcA...........................  20    1  seq
..............................................................cuccuuauaacgaucucaaggcC...........................  5     1  seq
..............................................................cuccuuauaacgaucucaaggcAa..........................  2     1  seq
...............................................................uccuuauaacgaucucaagg.............................  2     0  seq
...............................................................uccuuauaacgaucucaaggA............................  1     1  seq
...............................................................Cccuuauaacgaucucaaggc............................  1     1  seq
...............................................................uccuuauaacgaucucaaggc............................  29    0  seq
...............................................................uccuuauaacgaucucaaggcg...........................  13    0  seq
...............................................................uccuuauaacgaucucaaggcA...........................  5     1  seq
...............................................................uccuuauaacgaucucaaggcU...........................  81    1  seq
...............................................................uccuuauaacgaucucaaggcgU..........................  3     1  seq
................................................................cUuuauaacgaucucaaggc............................  1     1  seq
................................................................ccuuauaacgaucucaaggc............................  1     0  seq
................................................................ccuuauaacgaucucaaggcU...........................  31    1  seq
.................................................................cuuauaacgaucucaaggc............................  1     0  seq
...................................................................................cgaugggcgcggcacugc...........  16    0  seq
...................................................................................cgaugggcgcggcacugcA..........  1     1  seq
...................................................................................cgaugggcgcggcacugcg..........  1     0  seq
...................................................................................cgaugggcgcggcacugcgacu.......  12    0  seq
...................................................................................cgaugggcgcggcacugcgacuc......  2     0  seq
....................................................................................gaugggcgcggcacugcg..........  3     0  seq
....................................................................................gaugggcgcggcacugcgac........  1     0  seq
....................................................................................gaugggcgcggcacugcgacA.......  2     1  seq
....................................................................................gaugggcgcggcacugcgacu.......  13    0  seq
```

```
                    Mature                                    Star
gugauaugcccguuccucacguugugaccguuauaaugggcaugaagucauucugagcaaugcuccuuauaacgaucucaaggcgaugggcgcggcacugcgacucuugggc
....................................................................................gaugggcgcggcacugcgacuc......    6          0          seq
....................................................................................gaugggcgcggcacugcgacucuugggc    2          0          seq
```
